# Ruptured appendicitis with undiagnosed Graves’ disease: Contrast-induced impending thyroid storm

**DOI:** 10.1016/j.amsu.2022.104600

**Published:** 2022-09-09

**Authors:** Chutida Sungworawongpana, Wongsakorn Chaochankit

**Affiliations:** a Department of Anesthesiology, Faculty of Medicine, Prince of Songkla University, Songkhla, Thailand; b Department of Surgery, Faculty of Medicine, Prince of Songkla University, Songkhla, Thailand

**Keywords:** Appendicitis, Case report, Graves' disease, Hyperthyroidism, Iodine

## Abstract

**Background:**

Thyrotoxicosis is a generally common endocrine condition. Widespread radiologic studies and interventional treatments can increase the prevalence of contrast-induced thyrotoxicosis.

**Case presentation:**

A younger Thai female was diagnosed acute appendicitis that underwent computed tomography of abdomen with contrast injection. After the operation, she had fever and tachycardia without source of infection, but her thyroid function test showed hyperthyroidism, so she was diagnosed graves’ disease and received the treatment without thyroid storm.

**Discussion:**

Graves' disease is the most prevalent cause of hyperthyroidism in women but there are many causes of hyperthyroidism especially contrast-induced hyperthyroidism in this care, so the necessary of diagnostic tools especially contrast computed tomography should be carefully used in some patients.

**Conclusion:**

Critical radiologic studies and interventional procedures can increase the prevalence of contrast-induced thyrotoxicosis, particularly in high-risk patients. Utilizing a multidisciplinary team, clinicians must promptly address thyrotoxicosis and avert the thyroid storm.

## Highlights

• Thyrotoxicosis is a relatively common endocrine disorder.
• Thyrotoxicosis refers to the clinical symptoms of excess circulating thyroid hormones such as thyroiditis, and iodine-induced or drug-induced thyroid dysfunction.
• Diagnosis of thyrotoxicosis is upon clinical examination, laboratory tests, and further investigation to assist with diagnosing the specific cause and to arrange necessary treatment.
• The important issue among patients with thyrotoxicosis needing an operation is perioperative management and multidisciplinary care to prevent the thyroid storm that increases morbidity and mortality.

## 1 Introduction

Thyrotoxicosis is a moderately prevalent endocrine condition. The incidence of hyperthyroidism in the United States is 1.3% [1]. However, the conditions of hyperthyroidism and thyrotoxicosis vary. Thyrotoxicosis refers to the clinical signs of excess circulating thyroid hormones, including thyroiditis and iodine- or drug-induced thyroid malfunction. Graves' disease is the most prevalent cause of hyperthyroidism [2]. The diagnosis of thyrotoxicosis is based on a clinical examination, laboratory testing, and additional study to determine the precise cause and plan therapy. Patients with thyrotoxicosis who require surgery should get perioperative management and multidisciplinary care to prevent thyroid storm, increasing morbidity and mortality [1]. This case report revealed the main issue and caution of investigation in some patients that might be occurred some adverse events or complications from the diagnostic tools.

This case report has been reported in line with the SCARE Criteria [3].

## 2 Case presentation

A 19-year-old female presented with abdominal pain for three days. She came to the emergency department by herself. She had colicky pain around the umbilicus with a low-grade fever and then migratory pain for one day. She denied a history of past illness, family history illness or drug allergy. Her physical examination showed a normal appearance, and her lungs and heart were normal. Her abdomen revealed no distension, hypoactive bowel sound, and soft, moderate tenderness in the right lower quadrant area with localized guarding without mass. A completed blood count showed leukocytosis with a shift to the left (white blood cell 18350, PMN 81%), no anemia, and a normal platelet count. The urinalysis was normal and the urine pregnancy test showed negative. Her computed tomography of the abdomen revealed retrocecal acute appendicitis with a focal nonenhanced wall at the appendiceal tip. She received a diagnosis of gangrenous appendicitis and was sent to the operating theater for emergency laparoscopic appendectomy. The baseline vital signs included a blood pressure of 125/75 mmHg, a regular sinus rhythm heart rate of 120 beats/min, and peripheral oxygen saturation of 99%. Propofol (120 mg), succinylcholine (100 mg), and fentanyl (100 mcg) were administered intravenously, and the trachea was intubated using a 7.0 mm cuffed endotracheal tube. Anesthesia was maintained using 1 L/min O_2_, 1 L/min air, and 2.5–3.0 vol% sevoflurane. During the procedure, she presented persistent sinus tachycardia (heart rate 130–140 beats/min) and a fever (body temperature, 39 °C). Fever was treated using intravenous paracetamol 1000 mg and a cool pack. However, the E_T_CO_2_, maintained between 30 and 35 mmHg, had no signs of inadequate anesthesia or malignant hyperthermia. The anesthetist and surgeon discussed her tachycardia after treatment of dehydration and pyrexia. We suspected thyrotoxicosis and remained cautious regarding complications from this issue, e.g., the thyroid storm. The operation took approximately 1 hour and proceeded unremarkably. The neuromuscular blocker was then reversed using 2.5 mg neostigmine and 0.4 mg glycopyrrolate. Extubation was performed without complications. When the patient completely recovered from anesthesia, she was transferred to the PACU. In the ward, the patient presented BP of 140/100 mmHg, 100% SpO_2_, and the ECG showed sinus tachycardia with a HR of 140 beats/min. Complete physical exam found mild thyroid gland enlargement with thyroid built positive. She did not have palpitation or syncope. She had a history of unintentional weight loss of 17 kg over 4 months. Her thyroid function test revealed FT4 > 7.77 ng/dL (0.93–1.7), FT3 13.1 pg/mL (2–4.4), and TSH <0.005 mIU/L (0.27–4.2). The endocrinologist was consulted to co-evaluate and found the cause of severe thyrotoxicosis was suspected to be post iodine-contrast injection. The Burch Warsofsky point was 45 (impending storm) and thyroglobulin antibody (TgAb) was 16.1 IU/L (0–1.75). Her medications were PTU loading, then switched to methimazole, intravenous hydrocortisone total 1 day and propranolol. Her clinical conditions had been observed for two days, and she was discharged without complications. Her appendectomy wounds were completely healed, and the pathological report showed a ruptured appendix. The written informed consent for publication of the case details in this report has been obtained. At 6 months, her clinical conditions improved. Her weight was regained, and her thyroid function test showed FT4 5.02 ng/dL, FT3 13.3 pg/mL, and TSH <0.005 mIU/L. Her current medications were methimazole (5) 3 tabs per oral bid and propranolol (40) 1 tab po bid. She was followed up at the endocrine clinic regularly every 3 months. Six months after the first diagnosis of Graves’ disease, her FT4 was 0.46 ng/dL, FT3 2.77 pg/mL, and TSH 34.8 mIU/L respectively. Her clinicians stopped methimazole and consulted nuclear medicine team for radioactive iodine (RAI) (I-131) ingestion. She received RAI 15 mCi. Last follow up at endocrine clinic on July 2022, she did not have hyper- or hypothyroid function and her FT4 was 0.47 ng/dL, FT3 1.83 pg/mL, and TSH 71.8 mIU/L respectively. She received thyroxine (100) 1 tab per oral od and follow up next 1 month.

## 3 Discussion

Thyrotoxicosis is a prevalent symptom of endocrine disease [2]. Graves' disease is the most prevalent cause of hyperthyroidism in women between the ages of 20 and 40. Europe has a prevalence of 0.8% for hyperthyroidism, whereas the United States has a prevalence of 1.3%. There are several endogenous and extrinsic causes of thyrotoxicosis [1]. Exogenous thyrotoxicosis is caused by the consumption of large quantities of thyroid hormone and is characterized by low blood thyroglobulin concentrations. In this case report, the patient experienced various clinical symptoms associated with thyrotoxicosis [1]. Graves' disease was diagnosed due to her elevated TgAb level. It may explain why she was diagnosed with preclinical Graves' illness. She manifested clinical signs of hyperthyroidism after undergoing a CT scan of the abdomen with a contrast injection that resulted in excess contrast-induced thyrotoxicosis. It was discovered that this occurrence was hazardous since it might lead to a thyroid storm from preoperative to postoperative. Palpitations, dyspnea, and weight loss are clinical manifestations comparable to hyperthyroidism [4].

The mechanism of iodine- or contrast-induced thyrotoxicosis can be explained by extensive exposure of the thyroid gland to iodine [5]. Patients suspected of having iodine-induced thyrotoxicosis must undergo an iodine-contrast injection within 24 hours. Sodium-iodine symporters use iodine in the thyroid follicles to synthesize thyroid hormones [6]. Iodine at high concentrations can cause thyroid dysfunction. Diet, medicines, and iodine-contrast medium (ICM) for radiography are sources of iodine overload [7]. It has been observed that 300–500 g of iodide is sufficient to produce hyperthyroidism. A regular dosage of iodinated contrast media comprises approximately 13,500 g of free iodide and 15–60 g of bound iodine that can be released into the body [5]. Exogenous iodine reduces thyroidal radio-iodine absorption by diluting the total body iodine pool and reducing thyroid hormone production through the Wolff- Chaikoff effect. Exogenous iodine at high dosages inhibits organification of iodide and thyroid hormone production and may cause hypersecretion of thyroid hormones in people with euthyroidism. This phenomenon is known as the Jod–Basedow effect [8,9].

Nontoxic diffuse or nodular goiter, latent Graves' disease, and longstanding iodine deficiency are risk factors [10]. Patients suspected of ICM-induced thyrotoxicosis often have suppressed serum TSH values and high T4, FT4, and/or total T3 concentrations. Additional exposure to excess iodine should be avoided after diagnosing contrast-induced hyperthyroidism. We suspected iodine-induced thyrotoxicosis with undiagnosed Graves' illness, which TgAb verified in this instance. It is crucial to distinguish between the two kinds of thyrotoxicosis when a patient develops amiodarone- or iodine-induced thyrotoxicosis. When individuals with an underlying euthyroid nodular goiter or latent Graves' disease are exposed to the high iodine content of amiodarone, type I is typically the result. This exposure increases the synthesis and release of thyroid hormone, comparable to iodine-induced hyperthyroidism in individuals receiving excess iodine from other sources. It is treatable with antithyroid medications, but type II is damaging thyroiditis caused by amiodarone's direct toxic action on thyrocytes. This kind is often self-limiting, and amiodarone can be maintained if required [1].

In contrast, the Contrast Media Safety Committee of the European Society of Urogenital Radiology has determined that systematic monitoring of thyroid function prior to contrast injection in individuals with normal thyroid function is not necessary. However, high-risk individuals, i.e., those with a history of Graves' disease or nodular goiter, should be closely watched after undergoing iodinated contrast scans [11]. These include people who are primarily unable to tolerate thyroid malfunction, such as those with unstable cardiovascular illness. In the acute care context, CT scanning is becoming increasingly common. For example, between 2000 and 2005, chest CT scans increased by 226% in one emergency department, while abdominal CT scans increased by 72% [12]. A prompt diagnosis of thyrotoxicosis and knowledge of the frequency of intravenous contrast interfering with the use of radioactive iodine may save needless CT scans in specific individuals. Consequently, it may result in a higher incidence of ICM-induced thyrotoxicosis, severe thyrotoxicosis, or the thyroid storm. However, thyroid storm is an uncommon condition. In Japan, the incidence is 0.2 per 100,000 person-years, affecting 1–5% of hospitalized thyrotoxicosis patients. It is a medical emergency with a fatality rate ranging from 8% to 25% [13]. Thyroid storm's pathophysiology is still poorly understood. The clinical diagnosis is predicated on hyperthyroidism in a patient with life-threatening symptoms [1]. Burch and Wartofsky presented a scoring system for diagnosis, which was later updated by Akamizu and colleagues [13]. Utilize a multidisciplinary treatment approach. The treatment goals are to reduce the production and secretion of thyroid hormones, lower the number of thyroid hormones in the blood, control the effects of thyroid hormones on the body's edges, and treat any disease that worsens the condition [1].

## 4 Conclusion

The clinical signs of thyrotoxicosis continue to be very important. The objective of treatment for patients with thyrotoxicosis, mainly surgical patients, is to prevent a thyroid storm. Critical radiologic studies and interventional procedures can increase the prevalence of contrast-induced thyrotoxicosis, particularly in high-risk patients. Utilizing a multidisciplinary team, clinicians must promptly address thyrotoxicosis and avert the thyroid storm.

## Provenance and peer review

Not commissioned, externally peer reviewed.

## Ethical approval

The ethics committee of the Prince of Songkla University approved the protocol.

## Sources of funding

None.

## Author contributions

Study concept or design: Chutida Sungworawongpana, Wongsakorn Chaochankit.

Writing the manuscript: Chutida Sungworawongpana, Wongsakorn Chaochankit.

Review & editing the manuscript: Chutida Sungworawongpana, Wongsakorn Chaochankit.

## Registration of research studies

1 Name of the registry: None
2 Unique identifying number or registration ID: None
3 Hyperlink to your specific registration (must be publicly accessible and will be checked): None

## Guarantor

Wongsakorn Chaochankit.

## Consent

Written informed consent was obtained from the patient for publication of this case report.

## Declaration of competing interest

None.

## References

[1] De Leo S., Lee S.Y., Braverman L.E. (2016). Hyperthyroidism. Lancet..

[2] Hollowell J.G., Staehling N.W., Flanders W.D., Hannon W.H., Gunter E.W., Spencer C.A. (2002). Serum TSH, T_4_, and thyroid antibodies in the United States population (1988 to 1994): national health and nutrition examination survey (NHANES III). J. Clin. Endocrinol. Metab..

[3] Agha R.A., Franchi T., Sohrabi C., Mathew G., for the SCARE Group (2020). The SCARE 2020 guideline: updating consensus surgical CAse REport (SCARE) guidelines. Int. J. Surg..

[4] Devereaux D., Tewelde S.Z. (2014). Hyperthyroidism and thyrotoxicosis. Emerg. Med. Clin..

[5] Leung A.M., Braverman L.E. (2012). Iodine-induced thyroid dysfunction. Curr. Opin. Endocrinol. Diabetes Obes..

[6] Dai G., Levy O., Carrasco N. (1996). Cloning and characterization of the thyroid iodide transporter. Nature.

[7] Bizhanova A., Kopp P. (2009). Minireview: the sodium-iodide symporter NIS and pendrin in iodide homeostasis of the thyroid. Endocrinology.

[8] Dunne P., Kaimal N., MacDonald J., Syed A.A. (2013). Iodinated contrast-induced thyrotoxicosis. CMAJ (Can. Med. Assoc. J.).

[9] Zimmermann M.B. (2009). Iodine deficiency. Endocr. Rev..

[10] Lee S.Y., Rhee C.M., Leung A.M., Braverman L.E., Brent G.A., Pearce E.N. (2015). A review: radiographic iodinated contrast media-induced thyroid dysfunction. J. Clin. Endocrinol. Metab..

[11] Wu S.Y., Leung A.M., Chambers M.D., Chen C.L., Khan M.U., Brent G.A. (2018). Coexistent presentation of Graves' disease and an autonomous thyroid nodule following administration of an iodinated contrast load. Journal of Clinical and Translational Endocrinology: Case Reports.

[12] Phillips B.D., Hennessey J.V. (2009). Iodinated contrast prior to evaluation for thyrotoxicosis. J. Hosp. Med..

[13] Burch H.B., Wartofsky L. (1993). Life-threatening thyrotoxicosis. Thyroid storm. Endocrinol Metab. Clin. N. Am..

